# Acceptance and commitment therapy for chronic pain: A systematic review

**DOI:** 10.1192/j.eurpsy.2021.1166

**Published:** 2021-08-13

**Authors:** S. Freitas Ramos, A. Dias

**Affiliations:** 1 Department Of Psychiatry And Mental Health, Local Health Unit of Guarda, Guarda, Portugal; 2 Department Of Anesthesiology, Hospital Center Tâmega e Sousa, Penafiel, Portugal

**Keywords:** ACT, Chronic Pain

## Abstract

**Introduction:**

Chronic pain is common, costly, and associated with significant disability and negative effects on well-being and mental health. The treatment is challenging, requiring a multidisciplinary approach. Acceptance and commitment therapy (ACT) aims to help patients in engaging in a flexible and persistent pattern of values-directed behavior while in contact with continuing pain and discomfort.

**Objectives:**

To provide an updated review on the efficacy of ACT for the management of chronic pain.

**Methods:**

We conducted a systematic review based on the PubMed® and EBSCO databases up to April 2020.

**Results:**

Fifteen trials were included. The results were in favour of ACT in pain acceptance, functioning and pain intensity with small to large effect sizes. Few studies evaluated quality of life, but half of those were favourable to ACT. We also focused our analysis on ACT online interventions, considering the current demands due to the COVID-19 pandemic.

**Conclusions:**

The current systematic review points in favour of ACT for the management of chronic pain conditions, though the studies included suffered from methodological limitations, which may have led to overestimated effects. Methodologically robust trials are required to further understand the clinical efficacy of ACT for chronic pain and which patients most benefit from this intervention.

